# Supplementary material for: Metabolomics for predicting fetal growth restriction: protocol for a systematic review and meta-analysis
Source: BMJ Open. 2018 Dec 6;8(12):e022743. doi: 10.1136/bmjopen-2018-022743 (PMC6286473; doi:10.1136/bmjopen-2018-022743)
Supplement: Author's manuscript [file bmjopen-2018-022743.draft_revisions.pdf]

BMJ Open is committed to open peer review. As part of this commitment we make the peer review history of every article we publish publicly available.

When an article is published we post the peer reviewers' comments and the authors' responses online. We also post the versions of the paper that were used during peer review. These are the versions that the peer review comments apply to.

The versions of the paper that follow are the versions that were submitted during the peer review process. They are not the versions of record or the final published versions. They should not be cited or distributed as the published version of this manuscript.

BMJ Open is an open access journal and the full, final, typeset and author-corrected version of record of the manuscript is available on our site with no access controls, subscription charges or pay-per-view fees (http://bmjopen.bmj.com).

If you have any questions on BMJ Open's open peer review process please email info.bmjopen@bmj.com

BMJ Open

Metabolomics for predicting fetal growth restriction: protocol for a systematic review and meta-analysis

Journal:	*BMJ Open*
Manuscript ID	bmjopen-2018-022743
Article Type:	Protocol
Date Submitted by the Author:	07-Mar-2018

Complete List of Authors:	Leite, Debora; Campinas' State University, Department of Tocogynecology; Universidade Federal de Pernambuco,<br>Morillon, Aude-Claire; University College Cork National University of Ireland, Irish Centre for Fetal and Neonatal Translational Research (INFANT)<br>Melo Júnior, Elias; Universidade Federal de Pernambuco, Clinics Hospital<br>Souza, Renato; Universidade Estadual de Campinas, Obstetrics and Gynecology<br>Khashan, Ali; University College Cork, Department of Epidemiology and Public Health<br>Baker, Philip ; University of Leicester, College of Medicine<br>Kenny, Louise; University of Liverpool School of Life Sciences, Department of Women's and Children's Health<br>Cecatti, Jose Guilherme; University of Campinas, Obstetrics and Gynecology

Keywords:	fetal growth restriction, intrauterine growth restriction, small for gestational age infant, metabolomics, prediction

SCHOLARONE™
Manuscripts

Metabolomics for predicting fetal growth restriction: protocol for a systematic review and meta-analysis

Debora F. B. Leite[a,b] (deborafariasleite@gmail.com)
Aude-Claire Morillon[c] (audeclaire.morillon@ucc.ie)
Elias F. Melo Júnior[a] (eliasmelojr@gmail.com)
Renato T. Souza[b] (renatotsouzasp@gmail.com)
Ali S. Khashan[c,d] (a.khashan@ucc.ie)
Philip N. Baker[e] (philip.baker@leicester.ac.uk)
Louise C. Kenny[c,f] (louise.kenny@liverpool.ac.uk)
José Guilherme Cecatti[b] (cecatti@unicamp.br)

[a] Clinics Hospital of Federal University of Pernambuco, Recife, Pernambuco, Brazil

[b] Department of Gynaecology and Obstetrics, University Campinas, Campinas, Sao Paulo, Brazil

[c] Irish Centre for Fetal and Neonatal Translational Research (INFANT), University College Cork, Cork, Ireland.

[d] School of Public Health, University College Cork, Cork, Ireland

[e] College of Life Sciences, University of Leicester, England, United Kingdom.

[f] Faculty of Health and Life Sciences, Department of Women's and Children's Health, Institute of Translational Medicine, University of Liverpool, England, United Kingdom.

Corresponding author:
José Guilherme Cecatti
Rua Alexander Fleming, 101 Cidade Universitaria Zeferino Vaz.
Post code: 13083-889. Campinas, Sao Paulo, Brazil.
Telephone: +55 19-35219482
Email: cecatti@unicamp.br
Word count: 1,865

For peer review only

ABSTRACT

Introduction: fetal growth restriction (FGR) is a relevant research and clinical concern since it is related to higher risks of adverse outcomes at any period of life. Current predictive tools in pregnancy (clinical factors, ultrasound scan, placenta-related biomarkers) fail to identify the true growth-restricted fetus. However, technologies based on metabolomics have generated interesting findings, and seem promising. In this systematic review, we will address diagnostic accuracy of metabolomics analyses in predicting FGR.

Methods and analysis: Our primary outcome is small for gestational age infant (SGA), as a surrogate for FGR, defined as birth weight below the 10th centile by customized or population-based curves for gestational age. A detailed systematic literature search will be carried in electronic databases and conference abstracts, using the keywords 'fetal growth retardation', 'metabolomics', 'pregnancy', and 'screening' (and their variations). We will include original peer-reviewed articles published from 1998 till 2018, involving single pregnancies of fetuses without congenital malformations; sample collection must have been performed before clinical recognition of growth impairment. If additional information is required, authors will be contacted. Reviews, case reports, cross-sectional studies, non-human research, and commentaries papers will be excluded. Sample characteristics and the diagnostic accuracy data will be retrieved and analysed. If data allows, we will perform a meta-analysis.

Ethics and dissemination: As this is a systematic review, no ethical approval is necessary. This protocol will be publicized in our institutional websites, and results will be submitted for publication in a peer-reviewed journal.

Registration details: This protocol was registered at the PROSPERO platform with CRD42018089985.

For peer review only

Keywords: fetal growth restriction, intrauterine growth restriction, small for gestational age infant, metabolomics, prediction.

Strengths and limitations of this study

- This systematic review covers a great range of electronic databases and will also search for grey literature.
- Two researchers will perform literature search, data extraction, and study quality assessment independently, and any disagreement will be resolved by a third reviewer.
- Careful statistics procedures will be performed to identify accuracy of metabolomics in predicting fetal growth restriction.

For peer review only

INTRODUCTION

Fetal growth restriction (FGR) is usually defined as a fetus that has not reached its intrauterine growth potential, [1,2] with no major congenital abnormalities, [1] and has also been named as fetal growth retardation, intrauterine growth restriction or retardation (IUGR). [3] This heterogeneous condition is associated with increased risks of stillbirth, [4,5] neonatal intensive care unit admission, [6] neonatal mortality, [5] cognitive and behavioural impairment in infancy, [7] and chronic non transmissible disease in adulthood. [8] FGR is mainly diagnosed according to the estimated fetal weight in ultrasound scans below the 10th centile, [2,9] although it is anticipated that misdiagnosis can occur: fetuses below the 10th centile, but with normal outcomes ('constitutionally' small), or fetuses above the 10th centile, but who did not follow personal growth potential. [2] In this context, antenatal recognition of truly restricted fetuses, i.e. those at higher risk of morbidity and mortality in any period of life, followed by adequate obstetrical care, can improve neonatal outcomes. [10]

Unfortunately, in current practice, there is no gold standard for FGR diagnosis. Recent consensus has added ultrasound criteria (eg., abdominal circumference, umbilical and uterine artery Doppler measurements), and lowered estimated fetal weight cut-offs (<3rd centile), [1] to improve specificity. In these terms, the concept of FGR can overlap with that of small for gestational age (SGA), which includes infants with birth weight below the 10th (or 5th, or 3rd) centile for gender and gestational age. [11] In fact, it is common to use SGA as a surrogate for FGR. [6,12]

Since 1990's, when the thrifty phenotype theory was introduced, [13] a huge effort has been undertaken to investigate the pathologically growth restricted fetuses and newborns, and to enhance antenatal screening. [6] Clinical data has been intensively studied, with conflicting risk factors [14,15] and, in general, poor accuracy is achieved, even when first, [16] second [17,18] or third trimester [19] ultrasound parameters are added to prediction

For peer review only - http://bmjopen.bmj.com/site/about/guidelines.xhtml

model. Using only clinical or ultrasound variables, the great majority of SGA babies will only be recognized after birth, by population-based [5] or customised curves. [17] Biomarkers, such as placental growth factor (PlGF), soluble fms-like tyrosine kynase 1 (sFlt-1), and alfa-fetoprotein (AFP), [20] have each been found to show promise as aids to understanding FGR. However, the performance of these angiogenic factors as predictors of FGR has been limited (positive likelihood ratio, LR+, of 1.3 for PlGF and 1.4 for s-Flt-1). [21] Similarly, placental proteins are not robust enough biomarkers for FGR (for example, LR+ of 3.7 for placental protein-13 in first trimester). [22]) Therefore, there is a real need for better methods of FGR prediction.

The disappointing evidence may be due to the multifactorial nature of FGR; the aetiology of the condition is complex and poorly defined. Moreover, placental structure and functioning, maternal and fetal metabolism vary during pregnancy. [23] In this context, contemporary metabolomics approaches have identified several pathways and metabolic processes that may contribute to FGR, such as disruptions in DNA methylation, [24] cellular signalling, [25,26] neurotransmitter precursors, [26,27] and energy generation. [25,26]

Despite excellent performance of some metabolites in predicting FGR (area under the curve, AUC, above 0.9), [25,26] these studies have shown an overall modest accuracy. [21] However, only two 'omics' studies were included in Conde-Agudelo et al. [21] review, and issues related to gestational age of sampling and delivery, or analysis of composite outcomes, could have introduced bias and confounders to metabolomics findings. In recent years, many authors have applied diverse metabolomics techniques to predict FGR, suggesting that metabolite biomarkers may have a role to play in disease screening. Thus, the main objective of this systematic review will be to define the accuracy of metabolomics techniques for predicting FGR. As secondary aims, we will try to determine which metabolites are robust candidates for a prediction model of FGR, and which chemical class

they belong to.

METHODS AND ANALYSIS

Review question

What is the accuracy of metabolomics for predicting fetal growth restriction?

Condition or domain studied

Small for gestational age (SGA) infant. Fetal growth restriction (FGR).

Participants/ population

Inclusion criteria: Original studies including women with singleton gestation.

Exclusion: Multiple pregnancies. Congenital malformation.

Interventions/ exposure

Screening for SGA/FGR with metabolomics approach. Biomarker analysis should have been performed on samples taken before clinical recognition of neonatal outcome.

Inclusion and exclusion criteria

Original studies (cohort or case control studies) involving singleton pregnancies, as the studied population, and small for gestational age infant (and variations of terminology), as the outcome of interest, will be included in this systematic review.

The reasons for excluding studies are: (1) if they are Cross-sectional studies, Case Reports, Editorials, Letter to Editors, Commentaries, Expert Opinions, or any type of Reviews; (2) if they describe only experimental studies with animals; (3) if they show duplicate publication of the same data; in these cases, we will use the most recent publication.

Outcomes

Primary outcome: Small for gestational age infant, defined as a birth weight below the 10th centile according to population-based or to customized charts.

Secondary outcomes: birth weight below the 5th or the 3rd centile by population-based or customized parameters.

For peer review only - http://bmjopen.bmj.com/site/about/guidelines.xhtml

Literature search

The primary source of information will be these electronic databases: PubMed, EMBASE, Latin American and Caribbean Health Sciences Literature (LILACS), Scientific Electronic Library Online (Scielo), Health Technology Assessment (HTA), Database of Abstracts of Reviews of Effects (DARE), Aggressive Research Intelligence Facility (ARIF), Cumulative Index of Nursing and Allied Health Literature (CINAHL), Maternity and Infant Care (MIDIRS), Scopus, and Web of Science. Secondary sources include Google Scholar, hand-held searching of the reference list of eligible studies, conference proceedings, and contact with authors when necessary.

The keywords linked to the outcomes of interest will be combined with terms related to 'metabolomics' technique, 'pregnancy' and 'screening', using Boolean connectors. The same search strategy will be applied for each database, adapting for individual filters, main language, their own syntax and mechanisms of search; the complete search strategy is provided as Supplementary Material.

Studies published in the last twenty years (from 1998 to 2018) will be taken into account, and this systematic review will be performed from February, 2018. The search strategy will be re-run before final analysis, to check for recently published eligible studies. There are no language restrictions.

Data extraction and management

All searches will be exported to a reference manager (EndNote®). Individually, two researchers (DFBL and ACM) will select papers according to (i) title or abstract, and (ii) full text, that will be read only when abstracts are not sufficient to decide about inclusion criteria. Any disagreement about selected studies will be dealt by a third researcher (EFMJ or RTS); in these cases, only after majority decision (2:1 ratio) the next step will be performed. A fifth investigator (JGC) will revise all procedures before approving the Data Extraction. DFB, ACM and ASK will deal with the statistic procedures. JGC, PNB and LCK will re-examine all steps and supervise data interpretation.

A standardized form will be applied to extract the variables of interest, which will include: authors and year of publication, country of participants' enrolment, study design, definition used for FGR/SGA (customized or population-based charts) and outcome measured, number of affected (who later delivered a FGR/SGA baby) and non-affected pregnant women, gestational age of assessment, laboratory methods, and biological sample analysed (eg., blood, amniotic fluid). Researchers will contact authors (by electronic address)

For peer review only - http://bmjopen.bmj.com/site/about/guidelines.xhtml

if any clarification of data is needed. The metabolites described will be matched with the Human Metabolome database (HMDB), to check their biological function and chemical subclass.

Risk of bias assessment

Both investigators initially involved with literature search (DFBL and ACM) will assess methodological quality and applicability of all included studies, and they must check their judgements. A third researcher (EFMJ or RTS) will resolve any disagreement if necessary, and the final decisions will be made by majority. We will use the "Quality Assessment of Diagnostic Accuracy Studies" (QUADAS-2) [28] tool, which is comprised of four domains: patient selection, characteristics of index test (metabolomics technique), the reference standard test (measurement of birth weight), and flow and timing of patient inclusion and follow up. Every study will be labelled as 'low', 'high' or 'unclear' risk of bias for each domain. For example, there is 'low risk of bias' if the study clearly states how the metabolomics techniques were performed, or which birth weight curve was applied to identify the SGA babies.

Strategy for data synthesis

Details about data search and selection will be presented as a flow diagram, according to the Preferred Reporting Items for Systematic Reviews and Meta-Analysis (PRISMA) statement recommendations. [29] An aggregate participant data synthesis will be performed with all included studies; narrative data will be analysed and structured according to birth weight centile (10th, 5th, and 3rd) and curve type (population-based or customized curves). Studies characteristics and risk of bias assessment will be demonstrated in tables. Once possible, we will perform subgroup analysis according to:

- Which metabolomic methods were applied (gas or liquid chromatography coupled with mass spectrometry; or proton nuclear magnetic resonance);
- Maternal health status previous to pregnancy (healthy ones *versus* women with any chronic health condition).

Depending on data availability, accuracy measures will be calculated, and a meta-analysis will be drawn. Considering the quantitative nature of the metabolomics approach, and the expected different thresholds for metabolites in each study, we will try to perform the hierarchical summary receiver characteristic operating curve (HSROC). [30] Heterogeneity will also be assessed, through *I*-squared test.

Potential limitations to this review

Concerning the publication bias, we expect to encounter more published positive results, and data interpretation must take this issue in consideration. The metabolomics approach is highly detailed and meticulous, has shown great technological advancements in recent years, and results from mass spectrometry and from nuclear magnetic resonance complement each other. Therefore, we acknowledge that we may find distinct metabolites in each study, and generalisation may be challenging.

Possible confounders to interpret the selected studies will include clinical factors potentially associated to FGR/SGA, like parity, smoking habits, and previous history of fetal growth impairment. These characteristics will be considered during data extraction and synthesis.

Ethics and dissemination

As this is a systematic review protocol, no ethics committee approval is necessary. This protocol was registered in the International Prospective Register of Systematic Reviews (PROSPERO) database, registration number CRD42018089985, and follows the Preferred Reporting Items for Systematic Reviews and Meta-Analysis Protocols (PRISMA-P) statements. [31] A report of this systematic review will be sent to our sponsors. This protocol will be electronically available on UNICAMP-CNPq-Gates Foundation project website (www.medscinet.com/samba) and Infant Centre website (infantcentre.ie). Our results will be submitted to publication in peer-reviewed journal.

Patient and Public Involvement

Considering this is the protocol for a systematic review, patients and or public were not involved at all.

CONCLUSION

This systematic review will synthesize data about metabolomics and FGR/SGA, a promising field for understanding disease pathophysiology and natural history. By highlighting the metabolites, and chemical classes that they belong to, this review might present solid data to future research protocols, that can target the most promising compounds, or assess the participants in a more reliable gestational age, for example. A robust FGR/SGA

For peer review only

prediction assumes great importance in reproductive health and epidemiology, since this condition is associated with short and long-term adverse outcomes for the offspring.

Acknowledgements

We would like to thank Shauna Barret, librarian of Brookfield Library, University College Cork, Ireland, for her support with the literature search strategy. We are also grateful to Maeve O'Connell, for her suggestions to the final manuscript.

For peer review only

References

1 Gordijn SJ, Beune IM, Thilaganathan B, *et al.* Consensus definition of fetal growth restriction: a Delphi procedure. *Ultrasound Obstet Gynecol* 2016;48:333–9.

2 American College of Obstetricians and Gynecologists. ACOG Practice bulletin no. 134: fetal growth restriction. *Obstet Gynecol* 2013;121:1122–33.

3 Medical Subject Headings. https://www.ncbi.nlm.nih.gov/mesh/68005317 (accessed 23 Jan 2018).

4 Gardosi J, Madurasinghe V, Williams M, *et al.* Maternal and fetal risk factors for stillbirth: population based study. *BMJ* 2013;346:f108.

5 Mendez-Figueroa H, Truong VTT, Pedroza C, *et al.* Small-for-gestational-age infants among uncomplicated pregnancies at term: a secondary analysis of 9 Maternal-Fetal Medicine Units Network studies. *Am J Obstet Gynecol* 2016;215:628.e1–628.e7.

6 Sovio U, White IR, Dacey A, *et al.* Screening for fetal growth restriction with universal third trimester ultrasonography in nulliparous women in the Pregnancy Outcome Prediction (POP) study: a prospective cohort study. *Lancet* 2015;386:2089–97.

7 Korzeniewski SJ, Allred EN, Joseph RM, *et al.* Neurodevelopment at Age 10 Years of Children Born <28 Weeks With Fetal Growth Restriction. *Pediatrics* 2017;140. doi:10.1542/peds.2017-0697

8 Cheong JN, Wlodek ME, Moritz KM, *et al.* Programming of maternal and offspring disease: impact of growth restriction, fetal sex and transmission across generations. *J Physiol* 2016;594:4727–40.

9 Lausman A, Kingdom J. Intrauterine growth restriction: screening, diagnosis, and management. *J Obstet Gynaecol Can* 2013;35:741–8.

10 Verlijsdonk J-W, Winkens B, Boers K, *et al.* Suspected versus non-suspected small-for-gestational age fetuses at term: perinatal outcomes. *J Matern Fetal Neonatal Med* 2012;25:938–43.

11 Villar J, Cheikh Ismail L, Victora CG, *et al.* International standards for newborn weight, length, and head circumference by gestational age and sex: the Newborn Cross-Sectional Study of the INTERGROWTH-21st Project. *Lancet* 2014;384:857–68.

12 Malacova E, Regan A, Nassar N, *et al.* Risk of stillbirth, preterm delivery, and fetal growth restriction following exposure in a previous birth: systematic review and meta-analysis. *BJOG* 2018;125:183–92.

13 Hales CN, Barker DJ. The thrifty phenotype hypothesis. *Br Med Bull* 2001;60:5–20.

14 Abraham M, Alramadhan S, Iniguez C, *et al.* A systematic review of maternal smoking during pregnancy and fetal measurements with meta-analysis. *PLoS One*

For peer review only - http://bmjopen.bmj.com/site/about/guidelines.xhtml

2017;12:e0170946.

15 Vesterinen HM, Morello-Frosch R, Sen S, *et al.* Cumulative effects of prenatal-exposure to exogenous chemicals and psychosocial stress on fetal growth: Systematic-review of the human and animal evidence. *PLoS One* 2017;12:e0176331.

16 Velauthar L, Plana MN, Kalidindi M, *et al.* First-trimester uterine artery Doppler and adverse pregnancy outcome: a meta-analysis involving 55,974 women. *Ultrasound Obstet Gynecol* 2014;43:500–7.

17 McCowan LME, Thompson JMD, Taylor RS, *et al.* Clinical prediction in early pregnancy of infants small for gestational age by customised birthweight centiles: findings from a healthy nulliparous cohort. *PLoS One* 2013;8:e70917.

18 Parry S, Sciscione A, Haas DM, *et al.* Role of early second-trimester uterine artery Doppler screening to predict small-for-gestational-age babies in nulliparous women. *Am J Obstet Gynecol* 2017;217:594.e1–594.e10.

19 Tarca AL, Hernandez-Andrade E, Ahn H, *et al.* Single and Serial Fetal Biometry to Detect Preterm and Term Small- and Large-for-Gestational-Age Neonates: A Longitudinal Cohort Study. *PLoS One* 2016;11:e0164161.

20 Lesmes C, Gallo DM, Gonzalez R. *et al*. Prediction of small□for□gestational□age neonates: screening by maternal serum biochemical markers at 19–24 weeks. *Ultrasound Obstet Gynecol* 2015;46:341–9.

21 Conde-Agudelo A, Papageorghiou AT, Kennedy SH, *et al.* Novel biomarkers for predicting intrauterine growth restriction: a systematic review and meta-analysis. *BJOG* 2013;120:681–94.

22 Zhong Y, Zhu F, Ding Y. Serum screening in first trimester to predict pre-eclampsia, small for gestational age and preterm delivery: systematic review and meta-analysis. *BMC Pregnancy Childbirth* 2015;15:191.

23 Zhang S, Regnault TRH, Barker PL, *et al.* Placental adaptations in growth restriction. *Nutrients* 2015;7:360–89.

24 Horgan RP, Broadhurst DI, Dunn WB, *et al.* Changes in the Metabolic Footprint of Placental Explant-Conditioned Medium Cultured in Different Oxygen Tensions from Placentas of Small for Gestational Age and Normal Pregnancies. *Placenta* 2010;31:893–901.

25 Horgan RP, Broadhurst DI, Walsh SK, *et al.* Metabolic profiling uncovers a phenotypic signature of small for gestational age in early pregnancy. *J Proteome Res* 2011;10:3660–73.

26 Sulek K, Han T-L, Villas-Boas SG, *et al.* Hair metabolomics: identification of fetal compromise provides proof of concept for biomarker discovery. *Theranostics* 2014;4:953–9.

27 Maitre L, Fthenou E, Athersuch T, *et al.* Urinary metabolic profiles in early pregnancy are associated with preterm birth and fetal growth restriction in the Rhea mother--child

cohort study. *BMC Med* 2014;12:110.

28 Whiting PF, Rutjes AWS, Westwood ME, *et al.* QUADAS-2: a revised tool for the quality assessment of diagnostic accuracy studies. *Ann Intern Med* 2011;155:529–36.

29 Moher D, Liberati A, Tetzlaff J, *et al.* Preferred reporting items for systematic reviews and meta-analyses: the PRISMA statement. *PLoS Med* 2009;6:e1000097.

30 Leeflang MMG. Systematic reviews and meta-analyses of diagnostic test accuracy. *Clin Microbiol Infect* 2014;20:105–13.

31 Shamseer L, Moher D, Clarke M, *et al.* Preferred reporting items for systematic review and meta-analysis protocols (PRISMA-P) 2015: elaboration and explanation. *BMJ* 2015;349:g7647.

For peer review only

Contribution of authors

DFBL and ACM developed the systematic review protocol and will perform the literature search, study selection, data extraction, and risk of bias assessment. ASK, PNB, RTS, EFMJ, and JGC supervised protocol elaboration, and the latter three will resolve any discrepancy about methodology. ASK, DFBL and ACM will deal with statistics procedures. PNB and LCK performed the last amendments of protocol and will revise the final systematic review draft. All authors have read this manuscript and have agreed with this submission.

Funding

This research was supported by Brazilian National Research Council (grant number 401636/2013-5) and Bill and Melinda Gates Foundation (grant number OPP1107597- Grand Challenges Brazil: Reducing the burden of preterm birth, Fiotec number 05/2013), which provided funding to PRETERM-SAMBA project (www.medscinet.com/samba). DFBL was granted a scholarship (process number 88881.134512/2016-01) from the Brazilian Federal Agency for Support and Evaluation of Graduate Education (CAPES), and developed part of her doctoral studies as a visiting student at University College Cork, Ireland. RTS has also awarded a scholarship from CAPES (process number 88881.134095/2016-01). ACM was granted a scholarship from Science Foundation Ireland, for her doctoral thesis.

Competing interests

All authors are carrying original research or have published papers about metabolomics; DFBL and ACM are studying this technology in predicting FGR. JGC, LCK, and PNB have presented conference talks about this field. LCK and PNB are principal investigators of Metabolomic Diagnostics®.

For peer review only

Metabolomics for predicting fetal growth restriction: protocol for a systematic review and meta-analysis

	# Date
1	fetal growth retardation
2	fetal growth restriction
3	intrauterine growth restriction
4	intrauterine growth retardation
5	small for gestational age
6	#1 OR #2 OR #3 OR #4 OR #5
7	metabolomic*
8	metabonomic*
9	metabolit*
10	H NMR
11	proton NMR
12	proton nuclear magnetic resonance
13	liquid chromatogra*
14	gas chromatogra*
15	UPLC
16	ultra-performance liquid chromatograph*
17	ultra performance liquid chromatograph*
18	#7 OR #8 OR #9 OR #10 OR #11 OR #12 OR #13 OR #14 OR #15 OR #16 OR #17
19	pregnan*
20	antenat*
21	ante nat*
22	prenat*
23	pre nat*
24	#19 OR #20 OR #21 or #22 OR #23
25	screen*
26	predict*
27	metabolic profil*
28	#25 OR #26 OR #27
29	#6 AND #18 AND #24 AND 28

For peer review only

BMJ Open

Metabolomics for predicting fetal growth restriction: protocol for a systematic review and meta-analysis

Journal:	*BMJ Open*
Manuscript ID	bmjopen-2018-022743.R1
Article Type:	Protocol
Date Submitted by the Author:	11-Aug-2018

Complete List of Authors:	Leite, Debora; Campinas' State University, Department of Tocogynecology; Universidade Federal de Pernambuco,<br>Morillon, Aude-Claire; University College Cork National University of Ireland, Irish Centre for Fetal and Neonatal Translational Research (INFANT)<br>Melo Júnior, Elias; Universidade Federal de Pernambuco, Clinics Hospital<br>Souza, Renato; Universidade Estadual de Campinas, Obstetrics and Gynecology<br>Khashan, Ali; University College Cork, Department of Epidemiology and Public Health<br>Baker, Philip ; University of Leicester, College of Medicine<br>Kenny, Louise; University of Liverpool School of Life Sciences, Department of Women's and Children's Health<br>Cecatti, Jose Guilherme; University of Campinas, Obstetrics and Gynecology

**Primary Subject Heading**:	Obstetrics and gynaecology
Secondary Subject Heading:	Evidence based practice, Paediatrics
Keywords:	fetal growth restriction, intrauterine growth restriction, small for gestational age infant, metabolomics, prediction

SCHOLARONE™
Manuscripts

Metabolomics for predicting fetal growth restriction: protocol for a systematic review and meta-analysis

Debora F. B. Leite[a,b] (deborafariasleite@gmail.com)
Aude-Claire Morillon[c] (audeclaire.morillon@ucc.ie)
Elias F. Melo Júnior[a] (eliasmelojr@gmail.com)
Renato T. Souza[b] (renatotsouzasp@gmail.com)
Ali S. Khashan[c,d] (a.khashan@ucc.ie)
Philip N. Baker[e] (philip.baker@leicester.ac.uk)
Louise C. Kenny[c,f] (louise.kenny@liverpool.ac.uk)
José Guilherme Cecatti[b] (cecatti@unicamp.br)

[a] Clinics Hospital of Federal University of Pernambuco, Recife, Pernambuco, Brazil

[b] Department of Gynaecology and Obstetrics, University Campinas, Campinas, Sao Paulo, Brazil

[c] Irish Centre for Fetal and Neonatal Translational Research (INFANT), University College Cork, Cork, Ireland.

[d] School of Public Health, University College Cork, Cork, Ireland

[e] College of Life Sciences, University of Leicester, England, United Kingdom.

[f] Faculty of Health and Life Sciences, Department of Women's and Children's Health, Institute of Translational Medicine, University of Liverpool, England, United Kingdom.

Corresponding author:
José Guilherme Cecatti
Rua Alexander Fleming, 101 Cidade Universitaria Zeferino Vaz.
Post code: 13083-889. Campinas, Sao Paulo, Brazil.
Telephone: +55 19-3521-9482
Email: cecatti@unicamp.br

Word count: 2,038

For peer review only

ABSTRACT

Introduction: fetal growth restriction (FGR) is a relevant research and clinical concern since it is related to higher risks of adverse outcomes at any period of life. Current predictive tools in pregnancy (clinical factors, ultrasound scan, placenta-related biomarkers) fail to identify the true growth-restricted fetus. However, technologies based on metabolomics have generated interesting findings, and seem promising. In this systematic review, we will address diagnostic accuracy of metabolomics analyses in predicting FGR.

Methods and analysis: Our primary outcome is small for gestational age infant (SGA), as a surrogate for FGR, defined as birth weight below the 10th centile by customized or population-based curves for gestational age. A detailed systematic literature search will be carried in electronic databases and conference abstracts, using the keywords ‘fetal growth retardation’, ‘metabolomics’, ‘pregnancy’, and ‘screening’ (and their variations). We will include original peer-reviewed articles published from 1998 till 2018, involving pregnancies of fetuses without congenital malformations; sample collection must have been performed before clinical recognition of growth impairment. If additional information is required, authors will be contacted. Reviews, case reports, cross-sectional studies, non-human research, and commentaries papers will be excluded. Sample characteristics and the diagnostic accuracy data will be retrieved and analysed. If data allows, we will perform a meta-analysis.

Ethics and dissemination: As this is a systematic review, no ethical approval is necessary. This protocol will be publicized in our institutional websites, and results will be submitted for publication in a peer-reviewed journal.

Registration details: This protocol was registered at the PROSPERO platform with CRD42018089985.

Keywords: fetal growth restriction, intrauterine growth restriction, small for gestational age infant, metabolomics, prediction.

Strengths and limitations of this study

- This systematic review covers a great range of electronic databases and will also search for grey literature.
- Two researchers will perform literature search, data extraction, and study quality assessment independently, and any disagreement will be resolved by a third reviewer.
- Careful statistics procedures will be performed to identify accuracy of metabolomics in predicting fetal growth restriction.

For peer review only

INTRODUCTION

Fetal growth restriction (FGR) is usually defined as a fetus that has not reached its intrauterine growth potential, [1,2] with no major congenital abnormalities, [1] and has also been named as fetal growth retardation, intrauterine growth restriction or retardation (IUGR). [3] This heterogeneous condition is associated with increased risks of stillbirth, [4,5] neonatal intensive care unit admission, [6] neonatal mortality, [5] cognitive and behavioural impairment in infancy, [7] and chronic non transmissible disease in adulthood. [8] FGR is mainly diagnosed according to the estimated fetal weight in ultrasound scans below the 10th centile, [2,9] although it is anticipated that misdiagnosis can occur: fetuses below the 10th centile, but with normal outcomes ('constitutionally' small), or fetuses above the 10th centile, but who did not follow personal growth potential. [2] In this context, antenatal recognition of truly restricted fetuses, i.e. those at higher risk of morbidity and mortality in any period of life, followed by adequate obstetrical care, can improve neonatal outcomes. [10]

Unfortunately, in current practice, there is no gold standard for FGR diagnosis. Recent consensus has added ultrasound criteria (e.g., abdominal circumference, umbilical and uterine artery Doppler measurements), and lowered estimated fetal weight cut-offs (<3rd centile), [1] to improve specificity. In these terms, the concept of FGR can overlap with that of small for gestational age (SGA), which includes infants with birth weight below the 10th (or 5th, or 3rd) centile for gender and gestational age. [11] In fact, it is common to use SGA as a surrogate for FGR, [6,12,13] as an indication of real intrauterine growth impairment. Besides that, neonatal parameters seem more adequate as 'patient important outcomes', but regrettably, ultrasound have still low accuracy to determine them. [6]

Since 1990's, when the thrifty phenotype theory was introduced, [14] a huge effort has been undertaken to investigate the pathologically growth restricted foetuses and newborns, and to enhance antenatal screening. [6] Clinical data has been intensively studied,

For peer review only

with conflicting risk factors [15,16] and, in general, poor accuracy is achieved for identifying impaired birthweight [17] or neonatal morbidity, [6] even when first, [18] second [13,17] or third trimester [19] ultrasound parameters are added to prediction model. Using only clinical or ultrasound variables, the great majority of SGA babies will only be recognized after birth, by population-based [5] or customised curves. [17] Biomarkers, such as placental growth factor (PlGF), soluble fms-like tyrosine kynase 1 (sFlt-1), and alfa-fetoprotein (AFP), [20] have each been found to show promise as aids to understanding FGR. However, the performance of these angiogenic factors as predictors of FGR has been limited (positive likelihood ratio, LR+, of 1.3 for PlGF and 1.4 for s-Flt-1). [21] Similarly, placental proteins are not robust enough biomarkers for FGR (for example, LR+ of 3.7 for placental protein-13 in first trimester).[22]) Therefore, there is a real need for better methods of FGR prediction.

The disappointing evidence may be due to the multifactorial nature of FGR; the aetiology of the condition is complex and poorly defined. Moreover, placental structure and functioning, maternal and fetal metabolism vary during pregnancy. [23] In this context, contemporary metabolomics approaches have identified several pathways and metabolic processes that may contribute to FGR, such as disruptions in DNA methylation, [24] cellular signalling, [25,26] neurotransmitter precursors, [26,27] and energy generation. [25,26]

Despite excellent performance of some metabolites in predicting FGR (area under the curve, AUC, above 0.9), [25,26] these studies have shown an overall modest accuracy. [21] However, only two 'omics' studies were included in Conde-Agudelo et al. [21] review, and issues related to gestational age of sampling and delivery, or analysis of composite outcomes, could have introduced bias and confounders to metabolomics findings. In recent years, many authors have applied diverse metabolomics techniques to predict FGR, suggesting that metabolite biomarkers may have a role to play in disease screening. Thus, the main objective of this systematic review is to define the accuracy of metabolomics techniques

For peer review only - http://bmjopen.bmj.com/site/about/guidelines.xhtml

for predicting FGR. As secondary aims, we will try to determine which metabolites are robust candidates for a prediction model of FGR, and which chemical class they belong to.

METHODS AND ANALYSIS

Review question

What is the accuracy of metabolomics for predicting fetal growth restriction?

Condition or domain studied

Small for gestational age (SGA) infant. Fetal growth restriction (FGR).

Participants/ population

Inclusion criteria: Original studies including pregnant women.

Exclusion: Congenital malformation.

Interventions/ exposure

Screening for SGA/FGR with metabolomics approach. Biomarker analysis should have been performed on samples taken before clinical recognition of neonatal outcome.

Inclusion and exclusion criteria

Original studies (cohort or case control studies) involving pregnant women, as the studied population, and small for gestational age infant (and variations of terminology), as the outcome of interest, will be included in this systematic review.

The reasons for excluding studies are: (1) if they are Cross-sectional studies, Case Reports, Editorials, Letter to Editors, Commentaries, Expert Opinions, or any type of Reviews; (2) if they describe only experimental studies with animals; (3) if they show duplicate publication of the same data; in these cases, we will use the most recent publication.

Outcomes

Primary outcome: Small for gestational age infant, defined as a birth weight below the 10th centile according to population-based or to customized charts.

Secondary outcomes: birth weight below the 5th or the 3rd centile by population-based or customized parameters.

Literature search

The primary source of information will be these electronic databases: PubMed, EMBASE, Latin American and Caribbean Health Sciences Literature (LILACS), Scientific Electronic Library Online (Scielo), Health Technology Assessment (HTA), Database of Abstracts of Reviews of Effects (DARE), Aggressive Research Intelligence Facility (ARIF), Cumulative Index of Nursing and Allied Health Literature (CINAHL), Maternity and Infant Care (MIDIRS), Scopus, and Web of Science. Secondary sources include Google Scholar, hand-held searching of the reference list of eligible studies, conference proceedings, and contact with authors when necessary.

The keywords linked to the outcomes of interest will be combined with terms related to 'metabolomics' technique, 'pregnancy' and 'screening', using Boolean connectors. The same search strategy will be applied for each database, adapting for individual filters, main language, their own syntax and mechanisms of search; the complete search strategy is provided as Supplementary Material.

Considering that the term metabolome was first used in 1998 [28], we will take into account studies published in the last twenty years (1998 - 2018). The preliminary searches for this systematic review have started in February, 2018. The search strategy will be re-run before final analysis, to check for recently published eligible studies. There are no language restrictions.

Data extraction and management

All searches will be exported to a reference manager (EndNote®). Individually, two researchers (DFBL and ACM) will select papers according to (i) title or abstract, and (ii) full text, that will be read only when abstracts are not sufficient to decide about inclusion criteria. Any disagreement about selected studies will be dealt by a third researcher (EFMJ or RTS); in these cases, only after majority decision (2:1 ratio) the next step will be performed. A fifth investigator (JGC) will revise all procedures before approving the Data Extraction. DFBL, ACM and ASK will deal with the statistic procedures. JGC, PNB and LCK will re-examine all steps and supervise data interpretation.

A standardized form will be applied to extract the variables of interest – by two independent researchers - which will include: authors and year of publication, country of participants' enrolment, study design, definition used for FGR/SGA (customized or population-based charts) and outcome measured, number of affected (who later delivered a

FGR/SGA baby) and non-affected pregnant women, gestational age of assessment (throughout pregnancy), laboratory methods, and biological sample analysed (e.g., blood, amniotic fluid). In addition, data regarding growth impairment suspicion in pregnancy - such as gestational age, criteria applied for diagnosis, and follow up – will be retrieved once available. Researchers will contact authors (by electronic address) if any clarification of data is needed. The metabolites described will be matched with the Human Metabolome database (HMDB) to check their characteristics. [29]

Strategy for data synthesis

Details about data search and selection will be presented as a flow diagram, according to the Preferred Reporting Items for Systematic Reviews and Meta-Analysis (PRISMA) statement recommendations. [30] An aggregate participant data synthesis will be performed with all included studies; narrative data will be analysed and structured according to birth weight centile (10th, 5th, and 3rd) and curve type (population-based or customized curves). Additionally, the metabolites will be grouped and synthesized according to their biological function and chemical subclass. Studies' characteristics and risk of bias assessment will be demonstrated in tables. Once possible, we will perform subgroup analysis according to:

- Which metabolomic methods were applied (gas or liquid chromatography coupled with mass spectrometry; or proton nuclear magnetic resonance);
- Maternal health status before pregnancy (healthy ones *versus* women with any chronic health condition);
- Gestational age of first fetal growth impairment suspicion (early *versus* late FGR); [1]
- Type of pregnancy (single *versus* multiple).

Depending on data availability, accuracy measures will be calculated, and a meta-analysis will be drawn. Considering the quantitative nature of the metabolomics approach, and the expected different thresholds for metabolites in each study, we will try to perform the hierarchical summary receiver characteristic operating curve (HSROC). [31] Heterogeneity will also be assessed, through *I*-squared test.

Risk of bias assessment

Both investigators initially involved with literature search (DFBL and ACM) will assess methodological quality and applicability of all included studies, and they must check their judgements. A third researcher (EFMJ or RTS) will resolve any disagreement if necessary, and the final decisions will be made by majority. We will use the "Quality

Assessment of Diagnostic Accuracy Studies" (QUADAS-2) [32] tool, which is comprised of four domains: Patient Selection, characteristics of Index Test (metabolomics technique), the Reference Standard test (measurement of birth weight), and Flow and Timing of patient inclusion and follow up. Every study will be labelled as 'low', 'high' or 'unclear' risk of bias for each domain. For example, there is 'low risk of bias' if the study clearly states how the metabolomics techniques were performed, or which birth weight curve was applied to identify the SGA babies.

Regarding publication bias, we will assess the symmetry of funnel plots if more than ten studies are included in the meta-analysis. [33]

Potential limitations to this review

Concerning the publication bias, we expect to encounter more published positive results, and data interpretation must take this issue in consideration. The metabolomics approach is highly detailed and meticulous, has shown great technological advancements in recent years, and results from mass spectrometry and from nuclear magnetic resonance complement each other. Therefore, we acknowledge that we may find distinct metabolites in each study, and generalisation may be challenging.

In this systematic review, we have considered SGA as a proxy for FGR, as other authors. [6,12,13] The consensus for FGR diagnosis was published recently, [1] and past investigations may have used distinct terminology or conflicting criteria for this condition in pregnancy. Additional confounders to interpret the selected studies will include clinical factors potentially associated to FGR/SGA, like parity, smoking habits, and history of previous fetal growth impairment. These characteristics will be appraised during data extraction and synthesis, and detailed evidence will be retrieved.

Ethics and dissemination

As this is a systematic review protocol, no ethics committee approval is necessary. This protocol was registered in the International Prospective Register of Systematic Reviews (PROSPERO) database, registration number CRD42018089985, and follows the Preferred Reporting Items for Systematic Reviews and Meta-Analysis Protocols (PRISMA-P) statements. [34] A report of this systematic review will be sent to our sponsors. This protocol will be electronically available on UNICAMP-CNPq-Gates Foundation project website

(www.medscinet.com/samba) and Infant Centre website (infantcentre.ie). Our results will be submitted to publication in peer-reviewed journal.

Patient and Public Involvement

Patients and or public were not involved at all in elaborating this systematic review protocol.

CONCLUSION

This systematic review will synthesize data about metabolomics and FGR/SGA, a promising field for understanding disease pathophysiology and natural history. By highlighting the metabolites, and chemical classes that they belong to, this review might present solid data to future research protocols, that can target the most promising compounds, or assess the participants in a more reliable gestational age, for example. A robust FGR/SGA prediction assumes great importance in reproductive health and epidemiology, since this condition is associated with short and long-term adverse outcomes for the offspring.

Acknowledgements

We would like to thank Shauna Barret, librarian of Brookfield Library, University College Cork, Ireland, for her support with the literature search strategy. We are also grateful to Maeve O'Connell, for her suggestions to the final manuscript.

For peer review only

References

1 Gordijn SJ, Beune IM, Thilaganathan B, *et al.* Consensus definition of fetal growth restriction: a Delphi procedure. *Ultrasound Obstet Gynecol* 2016;**48**:333–9. doi:10.1002/uog.15884

2 American College of Obstetricians and Gynecologists (ACOG). Practice Bulletin 134. Fetal Growth Restriction. *Obstet Gynecol* 2013;**121**:1122–33.

3 Medical Subject Headings. https://www.ncbi.nlm.nih.gov/mesh/68005317 (accessed 23 Jan 2018).

4 Gardosi J, Madurasinghe V, Williams M, *et al.* Maternal and fetal risk factors for stillbirth: population based study. *Bmj* 2013;**346**:f108–f108. doi:10.1136/bmj.f108

5 Mendez-Figueroa H, Truong VTT, Pedroza C, *et al.* Small-for-gestational-age infants among uncomplicated pregnancies at term: a secondary analysis of 9 Maternal-Fetal Medicine Units Network studies. *Am J Obstet Gynecol* 2016;**215**:628.e1-628.e7. doi:10.1016/j.ajog.2016.06.043

6 Sovio U, White IR, Dacey A, *et al.* Screening for fetal growth restriction with universal third trimester ultrasonography in nulliparous women in the Pregnancy Outcome Prediction (POP) study: A prospective cohort study. *Lancet* 2015;**386**:2089–97. doi:10.1016/S0140-6736(15)00131-2

7 Korzeniewski SJ, Allred EN, Joseph RM, *et al.* Neurodevelopment at age 10 years of children born <28 weeks with fetal growth restriction. *Pediatrics* 2017;**140 (5) (n**. doi:http://dx.doi.org/10.1542/peds.2017-0697

8 Cheong JN, Wlodek ME, Moritz KM, *et al.* Programming of maternal and offspring disease: impact of growth restriction, fetal sex and transmission across generations. *J Physiol* 2016;**594**:4727–40. doi:10.1113/JP271745

9 Lausman A, Kingdom J, Gagnon R, *et al.* Intrauterine Growth Restriction: Screening, Diagnosis, And Management. *J Obstet Gynaecol Canada* 2013;**35**:741–8. doi:10.1016/S1701-2163(15)30865-3

10 Verlijsdonk JW, Winkens B, Boers K, *et al.* Suspected versus non-suspected small-for-gestational age fetuses at term: Perinatal outcomes. *J Matern Neonatal Med* 2012;**25**:938–43. doi:10.3109/14767058.2011.600793

11 Villar J, Ismail LC, Victora CG, *et al.* International standards for newborn weight, length, and head circumference by gestational age and sex: The Newborn Cross-Sectional Study of the INTERGROWTH-21st Project. *Lancet* 2014;**384**:857–68. doi:10.1016/s0140-6736(14)60932-6

12 Malacova E, Regan A, Nassar N, *et al.* Risk of stillbirth, preterm delivery, and fetal growth restriction following exposure in a previous birth: systematic review and meta-analysis. *BJOG An Int J Obstet Gynaecol* 2018;**125**:183–92. doi:10.1111/1471-0528.14906

13 Parry S, Sciscione A, Haas DM, *et al.* Role of early second-trimester uterine artery

For peer review only

Doppler screening to predict small-for-gestational-age babies in nulliparous women. *Am J Obstet Gynecol* 2017;**217**:594.e1-594.e10. doi:10.1016/j.ajog.2017.06.013

14 Hales CN, Barker DJP. The thrifty phenotype hypothesis: Type 2 diabetes. *Br Med Bull* 2001;**60**:5–20. doi:10.1093/bmb/60.1.5

15 Vesterinen HM, Morello-Frosch R, Sen S, *et al.* Cumulative effects of prenatal-exposure to exogenous chemicals and psychosocial stress on fetal growth: Systematic-review of the human and animal evidence. *PLoS One* 2017;**12**. doi:10.1371/journal.pone.0176331

16 Abraham M, Alramadhan S, Iniguez C, *et al.* A systematic review of maternal smoking during pregnancy and fetal measurements with meta-analysis. *PLoS One* 2017;**12**:1–13. doi:10.1371/journal.pone.0170946

17 McCowan LME, Thompson JMD, Taylor RS, *et al.* Clinical Prediction in Early Pregnancy of Infants Small for Gestational Age by Customised Birthweight Centiles: Findings from a Healthy Nulliparous Cohort. *PLoS One* 2013;**8**. doi:10.1371/journal.pone.0070917

18 Velauthar L, Plana MN, Kalidindi M, *et al.* First-trimester uterine artery Doppler and adverse pregnancy outcome: A meta-analysis involving 55 974 women. *Ultrasound Obstet Gynecol* 2014;**43**:500–7. doi:10.1002/uog.13275

19 Tarca AL, Hernandez-Andrade E, Ahn H, *et al.* Single and serial fetal biometry to detect preterm and term small- and large-for-gestational-age neonates: A longitudinal cohort study. *PLoS One* 2016;**11**:1–16. doi:10.1371/journal.pone.0164161

20 Poon LC, Lesmes C, Gallo DM, *et al.* Prediction of small-for-gestational-age neonates: Screening by biophysical and biochemical markers at 19-24 weeks. *Ultrasound Obstet Gynecol* 2015;**46**:437–45. doi:10.1002/uog.14904

21 Conde-Agudelo a, Papageorghiou a T, Kennedy SH, *et al.* Novel biomarkers for predicting intrauterine growth restriction: a systematic review and meta-analysis. *BJOG* 2013;**120**:681–94. doi:10.1111/1471-0528.12172

22 Zhong Y, Zhu F, Ding Y. Serum screening in first trimester to predict pre-eclampsia, small for gestational age and preterm delivery: Systematic review and meta-analysis. *BMC Pregnancy Childbirth* 2015;**15**:1–10. doi:10.1186/s12884-015-0608-y

23 Zhang S, Regnault TRH, Barker PL, *et al.* Placental adaptations in growth restriction. *Nutrients* 2015;**7**:360–89. doi:10.3390/nu7010360

24 Horgan RP, Broadhurst DI, Dunn WB, *et al.* Changes in the metabolic footprint of placental explant-conditioned medium cultured in different oxygen tensions from placentas of small for gestational age and normal pregnancies. *Placenta* 2010;**31**:893–901. doi:10.1016/j.placenta.2010.07.002

25 Horgan RP, Broadhurst DI, Walsh SK, *et al.* Metabolic profiling uncovers a phenotypic signature of small for gestational age in early pregnancy. *J Proteome Res* 2011;**10**:3660–73. doi:10.1021/pr2002897

For peer review only

26 Sulek K, Han TL, Villas-Boas SG ranat., *et al.* Hair metabolomics: identification of fetal compromise provides proof of concept for biomarker discovery. *Theranostics* 2014;**4**:953–9. doi:10.7150/thno.9265

27 Maitre L, Fthenou E, Athersuch T, *et al.* Urinary metabolic profiles in early pregnancy are associated with preterm birth and fetal growth restriction in the Rhea mother-child cohort study. *BMC Med* 2014;**12**:1–14. doi:10.1186/1741-7015-12-110

28 Oliver S, Böttcher C, Neumann S, *et al.* Systematic functional analysis of the yeast genome. *Trends Biotechnol* 1998;**16**:373–8. doi:10.1016/S0167-7799(98)01214-1

29 Wishart DS, Jewison T, Guo AC, *et al.* HMDB 3.0-The Human Metabolome Database in 2013. *Nucleic Acids Res* 2013;**41**:801–7. doi:10.1093/nar/gks1065

30 Moher D, Liberati A, Tetzlaff J, *et al.* Preferred Reporting Items for Systematic Reviews and Meta-Analyses: the PRISMA statement. *PLoS Med* 2009;**6**. doi:10.1371/journal.pmed1000097

31 Rutter CM, Gatsonis CA. A hierarchical regression approach to meta-analysis of diagnostic test accuracy evaluations. *Stat Med* 2001;**20**:2865–84. doi:10.1002/sim.942

32 Whiting PF, Rutjes AWS, Westwood ME, *et al.* Research and Reporting Methods Accuracy Studies. *Ann Intern Med* 2011;**155**:529–36. doi:10.7326/0003-4819-155-8-201110180-00009

33 Deeks JJ, Macaskill P, Irwig L. The performance of tests of publication bias and other sample size effects in systematic reviews of diagnostic test accuracy was assessed. *J Clin Epidemiol* 2005;**58**:882–93. doi:10.1016/j.jclinepi.2005.01.016

34 Shamseer L, Moher D, Clarke M, *et al.* Preferred reporting items for systematic review and meta-analysis protocols (prisma-p) 2015: Elaboration and explanation. *BMJ* 2015;**349**:1–25. doi:10.1136/bmj.g7647

For peer review only

Contribution of authors

DFBL (the guarantor of the review) and ACM developed the systematic review protocol and will perform the literature search, study selection, data extraction, and risk of bias assessment. ASK, PNB, RTS, EFMJ, and JGC supervised protocol elaboration, and the latter three will resolve any discrepancy about methodology. ASK, DFBL and ACM will deal with statistics procedures. PNB and LCK performed the last amendments of protocol and will revise the final systematic review draft. All authors have read this manuscript and have agreed with this submission.

Funding

This research was supported by Brazilian National Research Council (grant number 401636/2013-5) and Bill and Melinda Gates Foundation (grant number OPP1107597- Grand Challenges Brazil: Reducing the burden of preterm birth, Fiotec number 05/2013), which provided funding to PRETERM-SAMBA project (www.medscinet.com/samba). DFBL was granted a scholarship (process number 88881.134512/2016-01) from the Brazilian Federal Agency for Support and Evaluation of Graduate Education (CAPES), and developed part of her doctoral studies as a visiting student at University College Cork, Ireland. RTS has also awarded a scholarship from CAPES (process number 88881.134095/2016-01). ACM was granted a scholarship from Science Foundation Ireland, for her doctoral thesis. Our sponsors have not intervened in authors' decision to write the systematic review protocol or to submit this paper.

Competing interests

For peer review only

All authors are carrying original research or have published papers about metabolomics; DFBL and ACM are studying this technology in predicting FGR. JGC, LCK, and PNB have presented conference talks about this field. LCK and PNB are principal investigators of Metabolomic Diagnostics®.

For peer review only

Metabolomics for predicting fetal growth restriction: protocol for a systematic review and meta-analysis

#	Date
1	fetal growth retardation
2	fetal growth restriction
3	intrauterine growth restriction
4	intrauterine growth retardation
5	small for gestational age
6	#1 OR #2 OR #3 OR #4 OR #5
7	metabolomic*
8	metabonomic*
9	metabolit*
10	H NMR
11	proton NMR
12	proton nuclear magnetic resonance
13	liquid chromatogra*
14	gas chromatogra*
15	UPLC
16	ultra-performance liquid chromatograph*
17	ultra performance liquid chromatograph*
18	#7 OR #8 OR #9 OR #10 OR #11 OR #12 OR #13 OR #14 OR #15 OR #16 OR #17
19	pregnan*
20	antenat*
21	ante nat*
22	prenat*
23	pre nat*
24	#19 OR #20 OR #21 or #22 OR #23
25	screen*
26	predict*
27	metabolic profil*
28	#25 OR #26 OR #27
29	#6 AND #18 AND #24 AND 28

For peer review only

Metabolomics for predicting fetal growth restriction: protocol for a systematic review and meta-analysis

Debora F. B. Leite, Aude-Claire Morillon, Elias F. Melo Júnior, Renato T. Souza, Ali S. Khashan, Philip N. Baker, Louise C. Kenny, José Guilherme Cecatti

PRISMA-P checklist

Section and topic	Item	Checklist item	Page/ Lines
		**Administrative information**	
Title:			
Identification	1a	Identify the report as a protocol of a systematic review	01/ 01
Update	1b	If the protocol is for an update of a previous systematic review, identify as such	NA
Registration	2	If registered, provide the name of the registry (such as PROSPERO) and registration number	02/23
Authors:			
Contact	3a	Provide name, institutional affiliation, e-mail address of all protocol authors; provide physical mailing address of corresponding author	01/03-27
Contributions	3b	Describe contributions of protocol authors and identify the guarantor of the review	14/03-09
Amendments	4	If the protocol represents an amendment of a previously completed or published protocol, identify as such and list changes; otherwise, state plan for documenting important protocol amendments	NA
Support:			
Sources	5a	Indicate sources of financial or other support for the review	14/12-20
Sponsor	5b	Provide name for the review funder and/or sponsor	14/12-15
Role of sponsor or funder	5c	Describe roles of funder(s), sponsor(s), and/or institution(s), if any, in developing the protocol	14/20-22
		**Introduction**	
Rationale	6	Describe the rationale for the review in the context of what is already known	04-06
Objectives	7	Provide an explicit statement of the question(s) the review will address with reference to participants, interventions, comparators, and outcomes (PICO)	06/06
		**Methods**	
Eligibility criteria	8	Specify the study characteristics (such as PICO, study design, setting, time frame) and report characteristics (such as years considered, language, publication status) to be used as criteria for eligibility for the review	06/08-32
Information sources	9	Describe all intended information sources (such as electronic databases, contact with study authors, trial registers or other grey literature sources) with planned dates of coverage	07/02-20
Search strategy	10	Present draft of search strategy to be used for at least one electronic database, including planned limits, such that it could be repeated	07/11-15

For peer review only

Study records:			
Data management	11a	Describe the mechanism(s) that will be used to manage records and data throughout the review	07/23
Selection process	11b	State the process that will be used for selecting studies (such as two independent reviewers) through each phase of the review (that is, screening, eligibility and inclusion in meta-analysis)	07/23-30
Data collection process	11c	Describe planned method of extracting data from reports (such as piloting forms, done independently, in duplicate), any processes for obtaining and confirming data from investigators	07/31-32
Data items	12	List and define all variables for which data will be sought (such as PICO items, funding sources), any pre-planned data assumptions and simplifications	07/32-34 + 08/01-07
Outcomes and prioritization	13	List and define all outcomes for which data will be sought, including prioritization of main and additional outcomes, with rationale	08/09-23
Risk of bias in individual studies	14	Describe anticipated methods for assessing risk of bias of individual studies, including whether this will be done at the outcome or study level, or both; state how this information will be used in data synthesis	08/30-34 + 09/01-07
Data synthesis	15a	Describe criteria under which study data will be quantitatively synthesized	09/01-03
	15b	If data are appropriate for quantitative synthesis, describe planned summary measures, methods of handling data and methods of combining data from studies, including any planned exploration of consistency (such as I^2, Kendall's τ)	08/24-28
	15c	Describe any proposed additional analyses (such as sensitivity or subgroup analyses, meta-regression)	NA
	15d	If quantitative synthesis is not appropriate, describe the type of summary planned	08/23
Meta-bias(es)	16	Specify any planned assessment of meta-bias(es) (such as publication bias across studies, selective reporting within studies)	09/08-09
Confidence in cumulative evidence	17	Describe how the strength of the body of evidence will be assessed (such as GRADE)	NA

NA: not applicable.

For peer review only
